# Evaluation of the Effects of Acupuncture on Blood Flow in Humans with Ultrasound Color Doppler Imaging

**DOI:** 10.1155/2012/513638

**Published:** 2012-06-21

**Authors:** Shin Takayama, Masashi Watanabe, Hiroko Kusuyama, Satoru Nagase, Takashi Seki, Toru Nakazawa, Nobuo Yaegashi

**Affiliations:** ^1^Department of Traditional Asian Medicine, Graduate School of Medicine, Tohoku University, 2-1, Seiryo-machi, Aoba-ku, Sendai 980-8575, Japan; ^2^Department of Obstetrics and Gynecology, Tohoku University Graduate School of Medicine, Sendai 980-8574, Japan; ^3^Department of Ophthalmology and Visual Science, Graduate School of Medicine, Tohoku University, Sendai 980-8575, Japan

## Abstract

Color Doppler imaging (CDI) can be used to noninvasively create images of human blood vessels and quantitatively evaluate blood flow in real-time. The purpose of this study was to assess the effects of acupuncture on the blood flow of the peripheral, mesenteric, and retrobulbar arteries by CDI. Statistical significance was defined as *P* values less than 0.05. Blood flow in the radial and brachial arteries was significantly lower during needle stimulation on LR3 than before in healthy volunteers, but was significantly higher after needle stimulation than before. LR3 stimulation also resulted in a significant decrease in the vascular resistance of the short posterior ciliary artery and no significant change of blood flow through the superior mesenteric artery (SMA) during acupuncture. In contrast, ST36 stimulation resulted in a significant increase in blood flow through the SMA and no significant change in the vascular resistance of the retrobulbar arteries. Additionally, acupuncture at previously determined acupoints in patients with open-angle glaucoma led to a significant reduction in the vascular resistance of the central retinal artery and short posterior ciliary artery. Our results suggest that acupuncture can affect blood flow of the peripheral, mesenteric, and retrobulbar arteries, and CDI can be useful to evaluate hemodynamic changes by acupuncture.

## 1. Introduction

To date, no quantitative evaluation methods have been established for determining the physiological effectiveness of acupuncture. Therefore, researchers conduct experiments using a variety of approaches. In this study, we focused on the physiological reactions to acupuncture and investigated blood flow changes that result from acupuncture [1–5].

Many studies of acupuncture efficacy have been based on the results of animal experiments with anesthesia. These studies indicate that acupuncture works through physiological mechanisms that occur primarily in the autonomic nervous system [6–12]. When acupuncture is performed in human clinical practice, the conditions are very different from those in animal experiments. Additionally, because the invasive examination techniques that are often used to evaluate the results of acupuncture treatments affect the efficacy of those treatments, it is difficult to distinguish physiological reactions caused by acupuncture from those caused by the invasion necessary for examination. To determine the efficacy of acupuncture in humans, it is important that the examination method be noninvasive. We therefore used noninvasive color Doppler imaging (CDI) with ultrasound to evaluate blood flow. CDI is an examination technique that is widely used in the practice and research of Western medicine [13–21]. CDI can quantitatively measure intravascular blood flow in the extremities and in various organs in real-time. It is useful in the investigation of vessels, such as the peripheral, coronary, splenic, adrenal, and superior mesenteric arteries (SMA) [22]. In addition, the reproducibility of real-time and noninvasive hemodynamic measurement with CDI is reported elsewhere [23].

 In traditional Chinese medicine, LR3 (Taichong, located on the foot, 1.5–2 units above the web between the first and second toes [24]) is an acupoint on the liver meridian, which has the functions of “soothing the liver,” “regulating the blood,” and “opening into the eyes” [24]. We therefore hypothesized that LR3 acupuncture would affect hemodynamics in the peripheral arteries and the retrobulbar arteries. ST36 (Zusanli, located on the lower leg, 3 units below the lateral “eye” of the knee, approximately 1 finger width lateral to the tibia [24]), in contrast, is an acupoint on the stomach meridian, and is associated with the functions of gastrointestinal organs [25]. We therefore hypothesized that ST36 acupuncture would affect hemodynamics in the SMA. Because glaucoma prognosis and retrobulbar circulation are related [26–29], we also investigated the effects of acupuncture on retrobulbar circulation in open-angle glaucoma (OAG) patients. In this study, we introduce the noninvasive CDI with ultrasound to evaluate blood flow changes by acupuncture.

## 2. Materials and Methods

### 2.1. Ultrasound Technique for Blood Flow Measurement

We measured circulation in the upper limb, SMA, and retrobulbar vessels using an ultrasound system (Prosound *α*10; Aloka Co., Ltd, Tokyo, Japan). The system had a 13 MHz linear transducer and a 5 MHz convex transducer. We used the linear transducer to examine peripheral arteries and the retrobulbar vessels. We used the convex transducer to measure SMA circulation.

**Figure 1:**
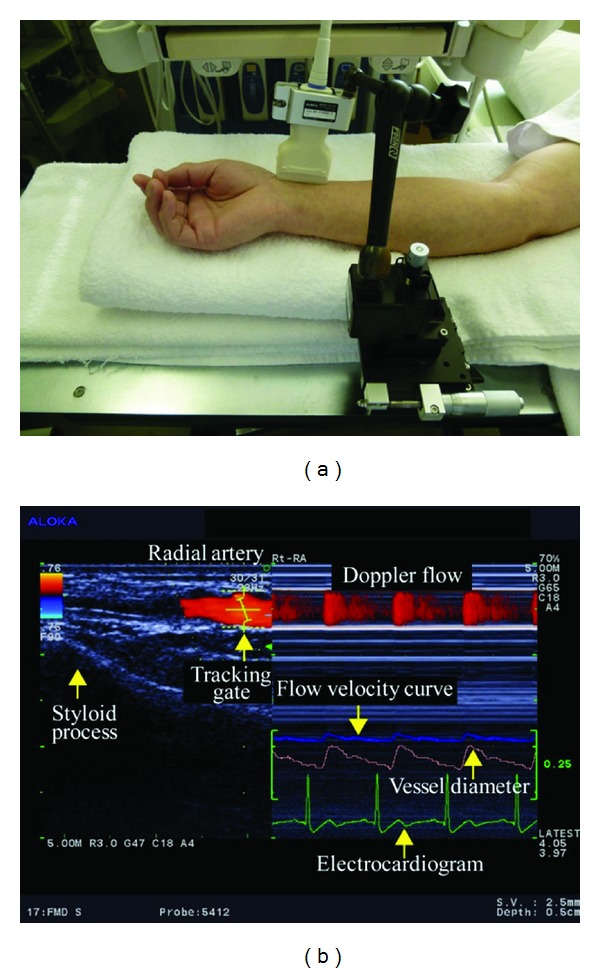
(a) Ultrasound measurement of the radial artery. 13 MHz linear transducer is fixed along radial artery with a special probe holder (MP-PH0001, Aloka Co., Ltd., Tokyo, Japan). (b) Display of CDI. Left: the vessel image and the position of the artery tracking gate. Right: changes in vessel diameter, Doppler flow, and flow velocity as determined by an automated edge-detection device and computer analysis software (e-Tracking system; Aloka Co., Ltd., Tokyo, Japan).

**Figure 2:**
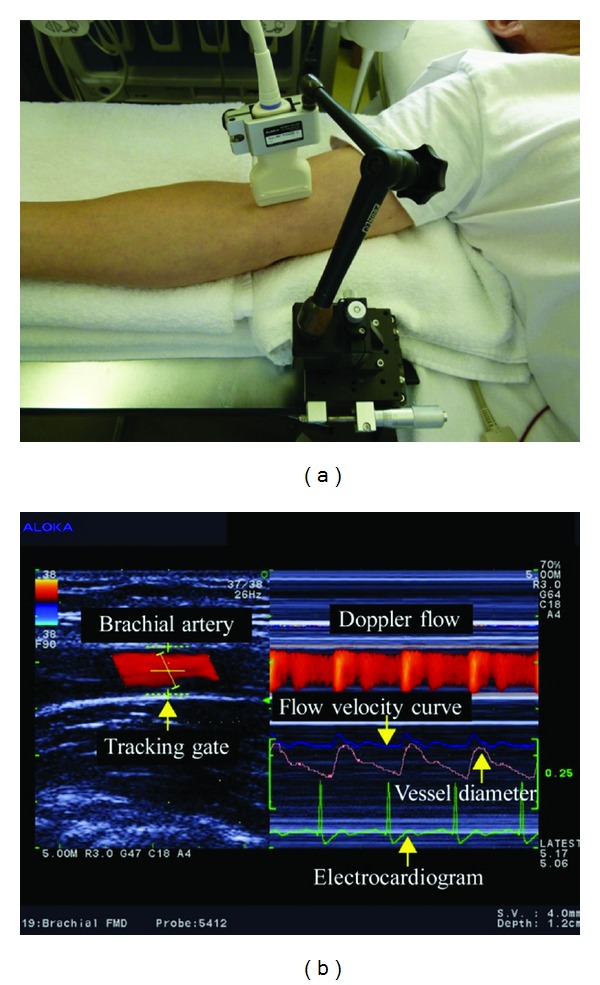
(a) Ultrasound measurement of the brachial artery. 13 MHz linear transducer is fixed along brachial artery with a special probe holder (MP-PH0001, Aloka Co., Ltd., Tokyo, Japan). (b) Display of CDI. Left: image of the vessel image and position of the artery tracking gate. Right: changes in vessel diameter, Doppler flow, and flow velocity, as determined by an automated edge detection device and computer analysis software (e-Tracking system; Aloka Co., Ltd., Tokyo, Japan).

**Figure 3:**
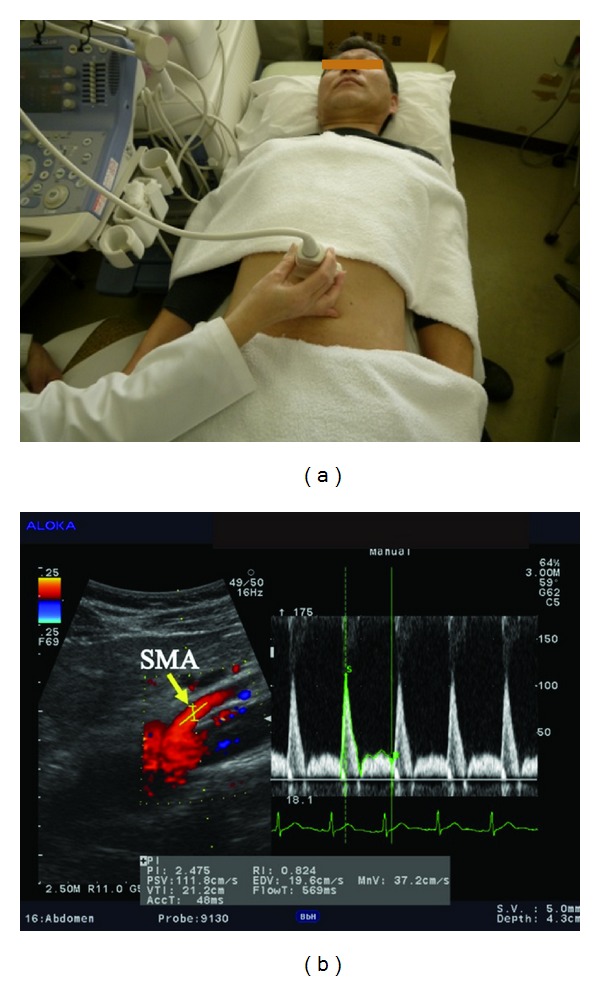
(a) Ultrasound measurement of the SMA. 5 MHz convex transducer is positioned on the abdomen. (b) Display of CDI. Left: image of the vessel and the position of the artery tracking. Right: Doppler flow and flow velocity.

**Figure 4:**
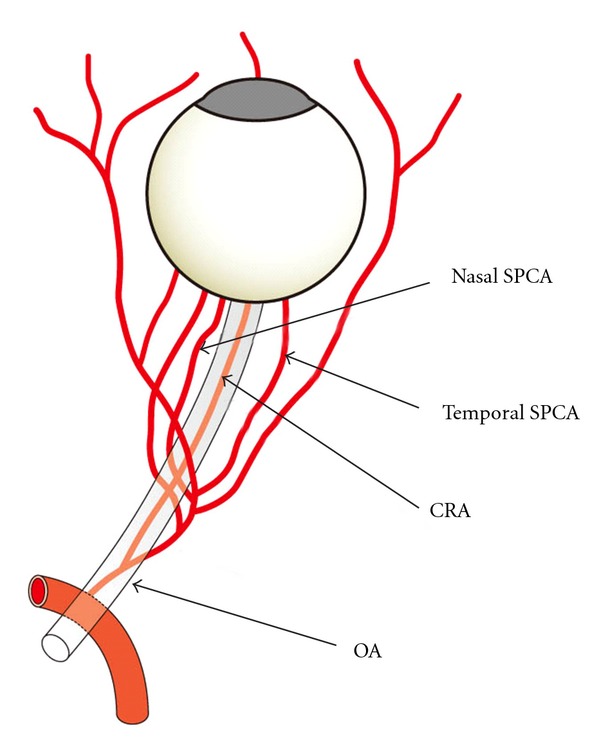
Schema of the retrobulbar arteries (OA: ophthalmic artery, CRA: central retinal artery, and SPCA: short posterior ciliary artery).

**Figure 5:**
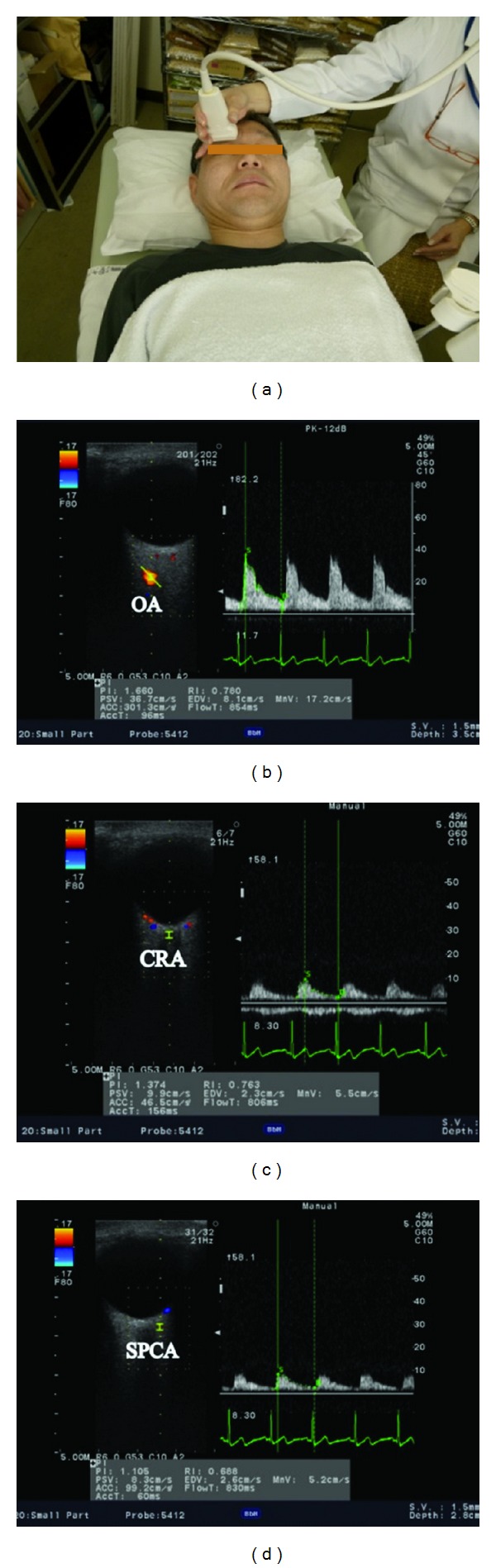
(a) Ultrasound measurement of retrobulbar arteries. 13 MHz linier transducer is attached on the eyelid. Horizontal scans by CDI through the ocular globe showing the (b) ophthalmic artery (OA), (c) central retinal artery (CRA), and (d) temporal short posterior ciliary artery (SPCA). Left: image of the vessel and the position of the artery tracking. Right: Doppler flow and flow velocity (b, c, and d).

 The radial artery was examined just medial to the radial styloid process (Figure 1). The brachial artery was monitored immediately proximal to the elbow (Figure 2). The SMA supplies blood to the whole small intestine, except for the superior part of the duodenum. It also supplies blood to the cecum, the ascending colon, and most of the transverse colon. SMA measurements were acquired within 2-3 cm of the artery origin (Figure 3) [30, 31]. Avoiding any pressure on the eye, CDI was performed for the retrobulbar vessels, including the ophthalmic artery (OA), central retinal artery (CRA), and nasal or temporal short posterior ciliary artery (Figures 4 and 5). The OA was examined approximately 20 mm behind the globe (Figure 5(b)), the CRA was examined within 5 mm of the retrolaminar portion of the optic nerve (Figure 5(c)), and the nasal or temporal SPCA that obtained clear image was examined approximately 5–10 mm behind the globe (Figure 5(d)). Blood flow was monitored continuously [32, 33] and we employed a Doppler angle of 60° or less for each measurement [34, 35]. Each Doppler waveform was automatically drawn and calculated using the software included with the ultrasound system. The following calculations were used to determine the hemodynamic parameters at each site [30, 31].

- Vessel diameter (VD).
- Cross-sectional area (CSA) = (VD/2)^2^ × *π*.
- Peak systolic velocity (PSV).
- End-diastolic velocity (EDV).
- Resistive index (RI) = (PSV − EDV)/PSV.
- Mean flow velocity (MV).
- Blood  flow  volume = CSA × MV.

### 2.2. Statistical Analysis

Statistical analysis was performed with SPSS software (version 16.0, SPSS Japan Inc., Tokyo, Japan). Repeated measure analysis of variance, followed by Dunnett's post hoc test, was used for statistical comparison between the measure points. Comparison between rest and after acupuncture was done by paired *t*-test. Results are presented as the mean ± SD and *P* < 0.05 was taken to indicate significance for all statistical analysis.

### 2.3. Experiment  1: Effects of LR3 Acupuncture on Upper Limb Circulation [1]

This study was employed to investigate the upper limb circulation after acupuncture at LR3 acupoints on foot. The participants were recruited by the poster recruitment in Tohoku University. Eighteen healthy volunteers (mean age:  32 ± 5  years; 14 males and 4 females) were enrolled in this study. A disposable fine stainless-steel needle (diameter: 0.16 mm; length: 40 mm; Seirin Co., Ltd., Shizuoka, Japan) was inserted on LR3 bilaterally and maintained at a depth of 10 mm during the test. After the needle was inserted, stimulation (rotating the needles manually within an angle of 90 degrees) was performed for 18 seconds. The needles were removed 200 seconds after acupuncture. Radial and brachial CDI were performed before acupuncture; during acupuncture treatment; 30, 60, and 180 seconds after acupuncture.

### 2.4. Experiment  2: Effects of LR3 Acupuncture on Blood Circulation to the Eye and through the SMA

This study was employed to clarify the hemodynamic changes by acupuncture in two different organs (intestine and eye) with simultaneous evaluation by ultrasound. The participants were recruited by the poster recruitment in Tohoku University. Thirteen healthy volunteers (mean age:  36 ± 9  years; 10 males and 3 females) were enrolled in this study. Acupuncture was performed bilaterally on LR3 with manual needle rotation and the disposable stainless steel needles (0.16 mm *× *40 mm; Seirin Co. Ltd., Shizuoka, Japan) were kept at the same site for 15 minutes. Retrobulbar vessels and SMA circulation were measured simultaneously at rest and 15 minutes after the start of acupuncture using ultrasound.

### 2.5. Experiment  3: Effects of ST36 Acupuncture Blood Circulation to the Eye and through the SMA

This study was also employed to clarify the hemodynamic changes by acupuncture in two different organs (intestine and eye) with simultaneous evaluation by ultrasound. The participants were recruited by the poster recruitment in Tohoku University. Thirteen subjects (mean age:  36 ± 8  years; 10 males and 3 females) were enrolled in this study. Acupuncture was performed bilaterally on ST36 with manual rotation of the disposable stainless steel needles (0.16 mm × 40 mm; Seirin Co. Ltd., Shizuoka, Japan) were kept in the same site for 15 minutes. Retrobulbar vessels and SMA circulation were measured simultaneously at rest and 15 minutes after the start of acupuncture using ultrasound.

### 2.6. Experiment  4: Effects of Acupuncture on Retrobulbar Circulation in OAG Patients [2]

The relation between glaucoma and retrobulbar circulation in the prognosis of the disease has been indicated [26–29], therefore we investigated the effects of acupuncture on OAG patients by CDI. The patients were recruited in the outpatient clinic of ophthalmology in Tohoku University Hospital. Eleven OAG patients (mean age:  63 ± 11  years; 1 male and 10 females; 20 eyes with OAG) were enrolled. All patients included in the study had been treated with topical antiglaucoma medications for at least 3 months prior to the study. As a control, the subjects received the measurements of retrobulbar vessel hemodynamics that were performed at rest and one hour after the first measurement. One month later, they received the same measurements before and after acupuncture treatment. Acupuncture was performed once bilaterally at acupoints BL2, EX-HN5, ST2, ST36, SP6, KI3, LR3, GB20, BL18, and BL23 for 15 minutes using disposable stainless steel needles (0.16 mm or 0.20 mm × 40 mm; Seirin Co. Ltd., Shizuoka, Japan). Retrobulbar circulation was measured using CDI at rest prior to treatment and 1 hour later, or after acupuncture.

## 3. Results and Discussion

### 3.1. Experiment  1: Effects of LR3 Acupuncture on Upper Limb Circulation [1]

**Figure 6:**
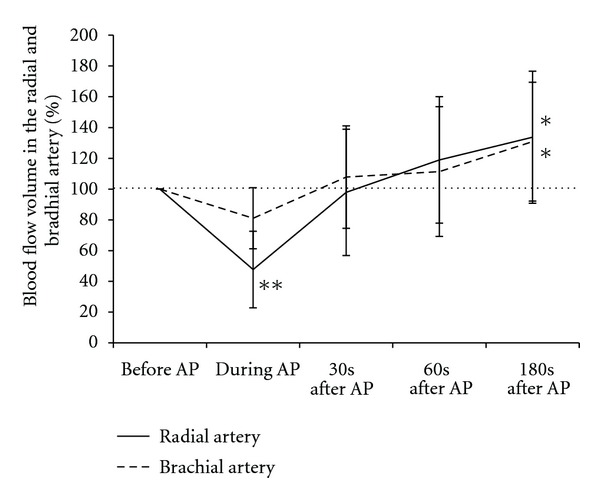
Percent changes in blood flow volume in the radial and brachial arteries before, during, and after acupuncture treatment. Values are presented as a percentage of the pretreatment blood flow. Values represent the mean and SD. AP: acupuncture. *P < 0.05, **P < 0.01 versus before acupuncture. Modified from [1].

Hemodynamic parameters including blood pressure, heart rate, and blood flow volume in the radial and brachial arteries are summarized in Table 1.  Figure 6 illustrates the profile of the percent changes in blood flow volume in the radial and brachial arteries. The blood flow volume in the radial artery decreased significantly during acupuncture (*P* < 0.01), but showed a significant increase at 180 seconds after acupuncture (*P* < 0.05) (Figure 6). In the brachial artery, the blood flow volume also showed a significant increase at 180 seconds after acupuncture (*P* < 0.05) (Figure 6). The physiological mechanisms of decrease and increase blood flow volume in upper limb are presumably related to a peripheral vascular resistance due to an instantaneous increase and decrease in sympathetic tone [1]. The present result suggests that LR3 located on the foot and apart from the upper limb can affect the circulation in the upper limb.

### 3.2. Experiment  2: Effects of LR3 Acupuncture on Blood Circulation to the Eye and through the SMA

The RI of the SPCA was significantly lower after acupuncture than before (*P* < 0.05; Table 2). However, blood flow volume in the SMA was not significantly changed after acupuncture than before (Table 2). The SPCA is the ocular branches of the OA and it supplies blood to the choroid (Figure 4) [32]. The decrease of the distal vascular resistance in the SPCA that we observed indicates that acupuncture on LR3 results in an increase of the blood flow to the choroid. It has been reported that the blood flow in the eye is controlled by sympathetic and parasympathetic nerves, and it is related with the release of nitric oxide or calcitonin gene-related peptide [33, 34]; it has also been reported that the regulation of regional blood flow by somatic afferent stimulation is based on somatoautonomic reflex mechanisms in the choroidal blood flow of the eyeball [34]. The hemodynamic changes in the SPCA by acupuncture may be related with these mechanisms. The present result suggests that LR3 located on the foot and apart from the eye can affect the circulation in the retrobulbar arteries.

### 3.3. Experiment  3: Effects of ST36 Acupuncture on Blood Circulation to the Eye and through the SMA

RI in the retrobulbar vessels was not changed by ST36 acupuncture treatment. However, the blood flow volume in the SMA was significantly greater after acupuncture than before (*P* < 0.05; Table 3). Acupuncture on the limbs was also demonstrated to elicit systemic visceral responses via the supraspinal reflexes in animal models [9, 36, 37]. According to several reports, blood flow volume in the SMA increased significantly after stimulation of the lower limbs [9, 36–38]. We speculate that this increase is caused by excitation of the parasympathetic system and inhibition of the sympathetic system via supraspinal reflexes. The present result suggests that ST36 located on the lower limb and apart from the abdomen can affect the circulation in the SMA.

### 3.4. Experiment  4: Effects of Acupuncture on Retrobulbar Circulation in OAG Patients

RI in the CRA and SPCA were significantly lower after acupuncture than it was before acupuncture treatment (CRA; *P* < 0.05, SPCA; *P* < 0.05; Table 4). RI in the SPCA was also significantly lower after acupuncture than when no treatment was given (SPCA; *P* < 0.01; Table 4). The CRA supplies blood to the retina and SPCA, to the choroid (Figure 4). The decrease of the distal vascular resistance in the CRA and SPCA that we observed indicates that acupuncture results in an increase of the blood flow to the retina and choroid. The possible physiological mechanisms of increase blood flow in eye has already described in the discussion of Experiment  2. The present result suggests that acupuncture can improve the retrobulbar circulation in the patients of OAG with standard medication.

## 4. Ultrasound and CDI

### 4.1. Advantage

 We focused on the evaluation of CDI by ultrasound. Noninvasive and real-time measure of CDI was applied to assess circulation in organs after acupuncture. The continuous method of CDI was used to assess the brief effects of circulation in the arm (Experiment  1). The simultaneous evaluation by CDI was applied to assess the circulation in two different organs (Experiments  2 and 3). Resistive index measured by CDI is measured in the small vessels as retrobulbar arteries (Experiment  4). Acupuncture affects the autonomic nervous system via the somatic nerves. Invasive evaluation also affects these systems and reflex. Therefore, invasive evaluation might not correctly evaluate the physiological effects of acupuncture therapy. We suggest that real-time and noninvasive hemodynamic measurement as CDI is suitable to measure the physiological effects in humans.

### 4.2. Limitation

 While CDI provides detailed images of blood vessels in real-time, there are limits to the hemodynamic measurements that can be made using this technique. In addition, while CDI is useful for the measurement of blood flow in various vessels in real time, it does not have sufficient resolution to determine the diameter of very small retrobulbar vessels such as OA, CRA, and SPCA. Therefore, CDI cannot be used to measure blood flow volume in these vessels. However, it can provide an index of vascular resistance such as RI. A decrease in the distal vascular resistance in the small vessels indicates an increase in the blood flow in the distal part of the vessels. Additionally, care must be taken to avoid compression of the eyeball during ultrasound examination. Such compression is likely to cause intraocular pressure elevation and trigger the vagal reflex. Measurement of blood flow in the retrobulbar arteries requires attention to probe maintenance and careful avoidance of pressure on the eyeball [23]. Expert technique is required to obtain reproducible results using CDI. In addition to the limits of CDI resolution, ultrasound waves that strike blood vessels at angles greater than 60° relative to the direction of blood flow result in a large margin of error for CDI measurements. Therefore, it is important to measure blood flow at a Doppler angle of less than 60 degrees [34, 35].

### 4.3. Further Study

The other methods to assess the physiological changes by acupuncture noninvasively are impedance cardiography and spectral analysis of heart rate variability. Impedance cardiography is a noninvasive monitoring method that allows measurement of the cardiac index based on the changes in thoracic resistance that results from variations in intrathoracic blood flow volume [39, 40]. Spectral analysis of heart rate variability is useful to evaluate the autonomic nervous balance noninvasively [41, 42]. Combined with these measurements, we can clarify the mechanism of increased blood flow volume in several organs in humans. In the future, we would like to explore the efficacy of acupuncture as treatment for various diseases by using diagnostic tools, such as CDI.

## 5. Conclusion

CDI can noninvasively depict blood vessels in the human body, and can quantitatively evaluate blood flow in real time. Our studies showed the changes of blood flow in the peripheral, mesenteric, and retrobulbar arteries by acupuncture estimated by CDI. This technique is suitable as an evaluation method to consider physiological changes due to acupuncture as blood flow changes.

## Figures and Tables

**Table 1:** Hemodynamic parameters and blood flow volume of the radial and brachial arteries by acupuncture on LR3. The values represent the mean and SD. *P < 0.05, **P < 0.01 versus before acupuncture. Modified from [1].

Parameters	Acupuncture on LR3				
	Before	During	30 s after	60 s after	180 s after
Systolic blood pressure (mmHg)	116.8 ± 10.1				114.5 ± 12.3
Diastolic blood pressure (mmHg)	67.3 ± 8.4				65.8 ± 7.3
Heart rate (beats/min)	67.3 ± 10.1	64.2 ± 8.8	65.8 ± 9.3	66.2 ± 9.3	66.9 ± 9.6
Blood flow volume of the radial artery (mL/min)	56.3 ± 33.5	25.4 ± 26.3	57.9 ± 47.5	67.7 ± 44.7	67.0 ± 36.5
Blood flow volume of the brachial artery (mL/min)	87.5 ± 56.4	65.7 ± 41.6	86.8 ± 53.7	90.1 ± 51.5	106.5 ± 59.8

**Table 2:** Hemodynamic parameters, blood flow volume of the SMA, and resistive index of retrobulbar arteries by acupuncture on LR3. The values represent the mean and SD. *P < 0.05, **P < 0.01 versus before acupuncture.

Parameters	Acupuncture on LR3	
	Before	After
Systolic blood pressure (mmHg)	119.6 ± 12.8	116.7 ± 11.1
Diastolic blood pressure (mmHg)	77.7 ± 9.4	76.5 ± 9.3
Heart rate (beats/min)	66.8 ± 7.1	63.3 ± 4.6**
Blood flow volume of the SMA (mL/min)	734.8 ± 312.9	704.4 ± 328.1
RI in OA	0.719 ± 0.097	0.707 ± 0.089
RI in CRA	0.661 ± 0.088	0.644 ± 0.052
RI in SPCA	0.624 ± 0.057	0.580 ± 0.037*

**Table 3:** Hemodynamic parameters, blood flow volume of the SMA, and resistive index of retrobulbar arteries by acupuncture on ST36. The values represent the mean and SD. *P < 0.05, **P < 0.01 versus before acupuncture.

Parameters	Acupuncture on ST36	
	Before	After
Systolic blood pressure (mmHg)	121.7 ± 11.8	120.7 ± 10.9
Diastolic blood pressure (mmHg)	77.8 ± 9.4	77.6 ± 7.6
Heart rate (beats/min)	61.9 ± 6.6	61.5 ± 7.4
Blood flow volume of the SMA (mL/min)	549.8 ± 192.2	620.2 ± 188.1*
RI in OA	0.736 ± 0.07	0.728 ± 0.070
RI in CRA	0.617 ± 0.065	0.631 ± 0.043
RI in SPCA	0.600 ± 0.030	0.580 ± 0.06

**Table 4:** Hemodynamic parameters and resistive index of retrobulbar arteries in control and acupuncture therapy. The values represent the mean and SD. *P < 0.05, **P < 0.01 versus rest or before acupuncture. ^†^ *P* < 0.05, ^††^ *P* < 0.01 versus control. Modified from [2].

Parameters	Control		Acupuncture	
	Rest	After 1 hour	Before	After
Systolic blood pressure (mmHg)	116.4 ± 10.0	119.8 ± 7.6	124.5 ± 12.9	122.6 ± 9.7
Diastolic blood pressure (mmHg)	69.8 ± 6.5	68.6 ± 3.9	74.5 ± 5.4	72.0 ± 2.9
Heart rate (beats/min)	61.5 ± 7.3	60.1 ± 8.1	61.7 ± 8.5	60.3 ± 10.4
RI in OA	0.74 ± 0.04	0.75 ± 0.05	0.74 ± 0.04	0.74 ± 0.04
RI in CRA	0.75 ± 0.09	0.72 ± 0.03	0.72 ± 0.05	0.68 ± 0.04*
RI in SPCA	0.68 ± 0.05	0.68 ± 0.04	0.67 ± 0.04	0.64 ± 0.06^∗††^
